# Supplementary material for: Gender-specific play behavior in relation to autistic traits and behavioral difficulties at the age of seven in the SELMA study
Source: PLoS One. 2024 Aug 28;19(8):e0308605. doi: 10.1371/journal.pone.0308605 (PMC11355531; doi:10.1371/journal.pone.0308605)
Supplement: S1 Table — (DOCX) [file pone.0308605.s001.docx]

S1 Table. Differences between the characteristics of included and excluded study participants.

|  | Included  (N=718) | Excluded  (N=243) | p value |
| --- | --- | --- | --- |
| Child age, mean in years, (SD)  *missing, N* | 7.5 (0.3)  0 | 7.6 (0.3)  3 | 0.03 |
| Mother age at birth, mean in years, (SD)  *missing, N* | 30.9 (4.7)  0 | 31.0 (4.6)  21 | 0.71 |
| Maternal education, N, (%)  Elementary school  High school  University  Other education  *missing, N* | 14 (1.9%)  197 (27.5%)  468 (65.2%)  39 (5.4%) | 5 (2.1%)  80 (32.9%)  124 (51.0%)  14 (5.8%)  20 (8.2%) | 0.08 |
|  | | |  |
| PSAI feminine subscore, mean, (SD)  *missing, N* | 24.9 (9.0)  0 | 24.0 (8.2)  98 | 0.25 |
| PSAI masculine subscore, mean, (SD)  *missing, N* | 27.5 (7.2)  0 | 28.0 (7.4)  101 | 0.51 |
| PSAI composite score, mean, (SD)  *missing, N* | 51.2 (14.8)  0 | 52.6 (14.9)  130 | 0.34 |
| Parental attitudes feminine subscore, mean, (SD)  *missing, N* | 20.9 (7.0)  0 | 20.4 (7.0)  113 | 0.42 |
| Parental attitudes masculine subscore, mean, (SD)  *missing, N* | 21.2 (6.6)  0 | 21.4 (7.0)  109 | 0.72 |
|  | | |  |
| SRS, total T-score, mean, (SD)  *missing, N* | 45.8 (8.3)  0 | 46.1 (7.9)  56 | 0.64 |
| SRS, social awareness, mean, (SD)  *missing, N* | 48.8 (10.1)  0 | 48.5 (9.9)  54 | 0.78 |
| SRS, social cognition, mean, (SD)  *missing, N* | 45.3 (8.7)  0 | 45.8 (9.3)  55 | 0.56 |
| SRS, social communication, mean, (SD)  *missing, N* | 45.5 (7.7)  0 | 46.1 (7.3)  56 | 0.34 |
| SRS, social motivation, mean, (SD)  *missing, N* | 48.1 (7.9)  0 | 48.1 (7.5)  55 | 0.91 |
| SRS, RIRB, mean, (SD)  *missing, N* | 45.8 (8.7)  0 | 45.9 (9.4)  53 | 0.22 |
|  |  |  |  |
| SDQ, total score, mean, (SD)  *missing, N* | 5.4 (4.6)  0 | 6.0 (5.2)  64 | 0.59 |
| SDQ, emotional symptoms, mean, (SD)  *missing, N* | 1.5 (1.7)  0 | 1.4 (1.8)  62 | 0.49 |
| SDQ, conduct problems, mean, (SD)  *missing, N* | - 1. (1.3)   0 | - 1. (1.5)   62 | 0.79 |
| SDQ, hyperactivity/inattention, mean, (SD)  *missing, N* | 2.4 (2.3)  0 | 2.7 (2.5)  63 | 0.26 |
| SDQ, peer relationships problems, mean, (SD)  *missing, N* | 0.7 (1.2)  0 | 0.7 (1.3)  63 | 0.82 |
| SDQ, prosocial behavior, mean, (SD)  *missing, N* | 8.8 (1.5)  0 | 8.8 (1.5)  62 | 0.77 |
