# Supplementary material for: Gender-specific play behavior in relation to autistic traits and behavioral difficulties at the age of seven in the SELMA study
Source: PLoS One. 2024 Aug 28;19(8):e0308605. doi: 10.1371/journal.pone.0308605 (PMC11355531; doi:10.1371/journal.pone.0308605)
Supplement: S2 Table — (DOCX) [file pone.0308605.s002.docx]

S2 Table. Unadjusted associations between gender-specific play behavior scores and autistic traits (N=718).

|  | SRS  total score | Social  awareness | Social  cognition | Social  communication | Social  motivation | RIRB |
| --- | --- | --- | --- | --- | --- | --- |
|  | beta (95% CI) | | | | | |
| Girls | | | | | | |
| Feminine score | -0.09 (-0.23, 0.05) | -0.15 (-0.33, 0.04) | -0.08 (-0.22, 0.07) | -0.08 (-0.21, 0.05) | -0.06 (-0.19, 0.07) | -0.06 (-0.22, 0.09) |
| Masculine score | 0.02 (-0.14, 0.18) | 0.08 (-0.13, 0.28) | 0.02 (-0.15, 0.18) | -0.02 (-0.17, 0.13) | -0.01 (-0.16, 0.13) | 0.06 (-0.11, 0.23) |
| Composite score | 0.07 (-0.04, 0.17) | 0.13 (-0.01, 0.27) | 0.06 (-0.05, 0.17) | 0.04 (-0.06, 0.14) | 0.03 (-0.06, 0.13) | 0.07 (-0.05, 0.18) |
| Boys | | | | | | |
| Feminine score | -0.05 (-0.25, 0.15) | -0.08 (-0.31, 0.15) | -0.10 (-0.31, 0.11) | -0.12 (-0.30, 0.07) | 0.12 (-0.08, 0.32) | 0.01 (-0.20, 0.21) |
| Masculine score | 0.05 (-0.10, 0.19) | 0.05 (-0.11, 0.22) | 0.04 (-0.11, 0.20) | -0.00 (-0.14, 0.13) | 0.02 (-0.12, 0.16) | 0.09 (-0.06, 0.23) |
| Composite score | 0.07 (-0.07, 0.20) | 0.09 (-0.06, 0.23) | 0.09 (-0.05, 0.22) | 0.05 (-0.07, 0.17) | -0.04 (-0.17, 0.09) | 0.08 (-0.06, 0.21) |

SRS, Social Responsiveness Scale; RIRB, Restricted interests and repetitive behavior; CI, confidence interval
