# Supplementary material for: Gender-specific play behavior in relation to autistic traits and behavioral difficulties at the age of seven in the SELMA study
Source: PLoS One. 2024 Aug 28;19(8):e0308605. doi: 10.1371/journal.pone.0308605 (PMC11355531; doi:10.1371/journal.pone.0308605)
Supplement: S3 Table — (DOCX) [file pone.0308605.s003.docx]

S3 Table. Unadjusted associations between gender-specific play behavior scores and behavioral outcomes (N=718).

|  | SDQ  total score | Emotional  symptoms | Conduct problems | Hyperactivity/ inattention | Peer relationships problems | Prosocial behavior |
| --- | --- | --- | --- | --- | --- | --- |
|  | beta (95% CI) | | | | | |
| Girls | | | | | | |
| Feminine score | -0.06 (-0.13, 0.02) | 0.00 (-0.03, 0.03) | **-0.03 (-0.05, -0.01)** | -0.02 (-0.06, 0.02) | -0.01 (-0.03, 0.01) | **0.04 (0.02, 0.07)** |
| Masculine score | 0.08 (-0.01, 0.16) | -0.01 (-0.04, 0.03) | 0.02 (-0.01, 0.05) | **0.05 (0.00, 0.09)** | 0.02 (-0.01, 0.04) | -0.00 (-0.03, 0.03) |
| Composite score | **0.07 (0.02, 0.13)** | -0.01 (-0.03, 0.02) | **0.03 (0.01, 0.05)** | **0.04 (0.01, 0.07)** | **0.01 (0.00, 0.03)** | **-0.03 (-0.05, -0.01)** |
| Boys | | | | | | |
| Feminine score | 0.00 (-0.11, 0.11) | 0.01 (-0.03, 0.05) | -0.02 (-0.05, 0.01) | -0.01 (-0.07, 0.05) | 0.03 (-0.00, 0.06) | 0.04 (0.01, 0.08) |
| Masculine score | **0.09 (0.01, 0.17)** | 0.02 (-0.01, 0.05) | 0.01 (-0.01, 0.03) | **0.05 (0.01, 0.09)** | 0.01 (-0.01, 0.03) | 0.02 (-0.01, 0.05) |
| Composite score | **0.08 (0.01, 0.15)** | 0.01 (-0.01, 0.04) | 0.02 (-0.00, 0.04) | **0.05 (0.01, 0.08)** | -0.00 (-0.02, 0.02) | -0.00 (-0.03, 0.02) |

SDQ, Strengths and Difficulties Questionnaire; CI, confidence interval
