# Supplementary material for: Gender-specific play behavior in relation to autistic traits and behavioral difficulties at the age of seven in the SELMA study
Source: PLoS One. 2024 Aug 28;19(8):e0308605. doi: 10.1371/journal.pone.0308605 (PMC11355531; doi:10.1371/journal.pone.0308605)
Supplement: S4 Table — (DOCX) [file pone.0308605.s004.docx]

S4 Table. Unadjusted associations between gender-specific play behavior scores and autistic traits and clinically relevant behavioral outcomes (N=718).

|  | SRS lower cut-off | SRS higher cut-off | SDQ 90th percentile |
| --- | --- | --- | --- |
|  | OR (95% CI) | | |
| Girls | | | |
| Feminine score | 0.93 (0.86, 1.00) | 0.87 (0.72, 1.05) | **0.94 (0.89, 0.99)** |
| Masculine score | 0.98 (0.90, 1.07) | 1.00 (0.81, 1.24) | 1.01 (0.95, 1.08) |
| Composite score | 1.04 (0.98, 1.09) | 1.08 (0.95, 1.22) | **1.04 (1.00, 1.09)** |
| Boys | | | |
| Feminine score | 0.99 (0.89, 1.10) | 1.03 (0.87, 1.22) | 1.04 (0.97, 1.12) |
| Masculine score | 1.01 (0.94, 1.08) | 1.05 (0.93, 1.18) | 1.04 (0.99, 1.10) |
| Composite score | 1.01 (0.94, 1.08) | 1.03 (0.92, 1.15) | 1.02 (0.97, 1.07) |

SRS, Social Responsiveness Scale; SDQ, Strengths and Difficulties Questionnaire; OR, odds ratio; CI, confidence interval
