# Supplementary figures and images for: Gender-specific play behavior in relation to autistic traits and behavioral difficulties at the age of seven in the SELMA study
Source: PLoS One. 2024 Aug 28;19(8):e0308605. doi: 10.1371/journal.pone.0308605 (PMC11355531; doi:10.1371/journal.pone.0308605)

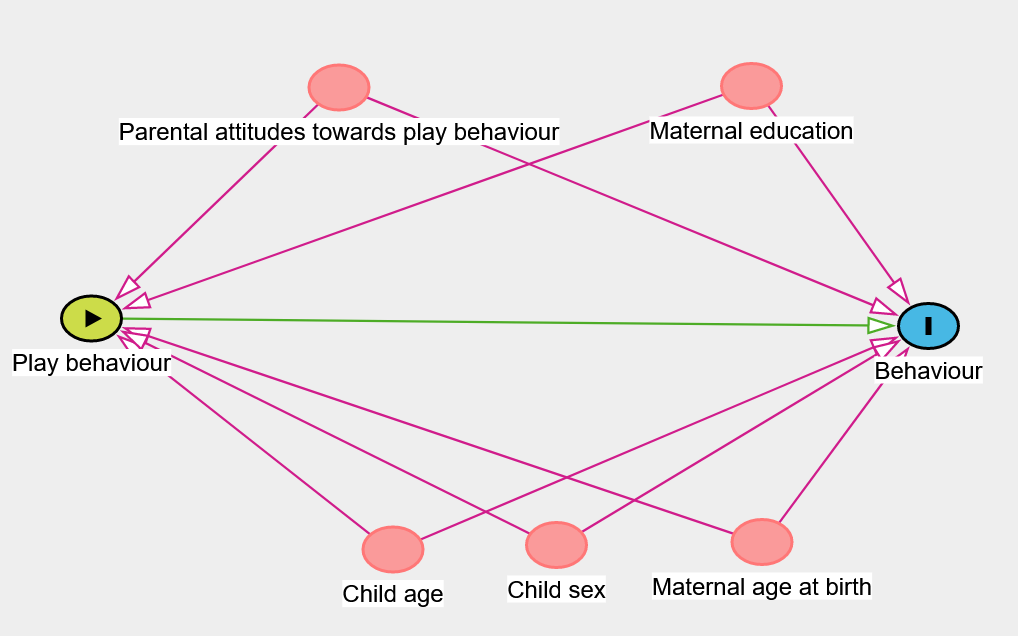

Supplement: S1 Fig — The green node indicates the independent variable, the blue node the dependent variable. The red nodes indicate potential confounding factors. I indicates the dependent variable. (TIF) [file pone.0308605.s005.tif]

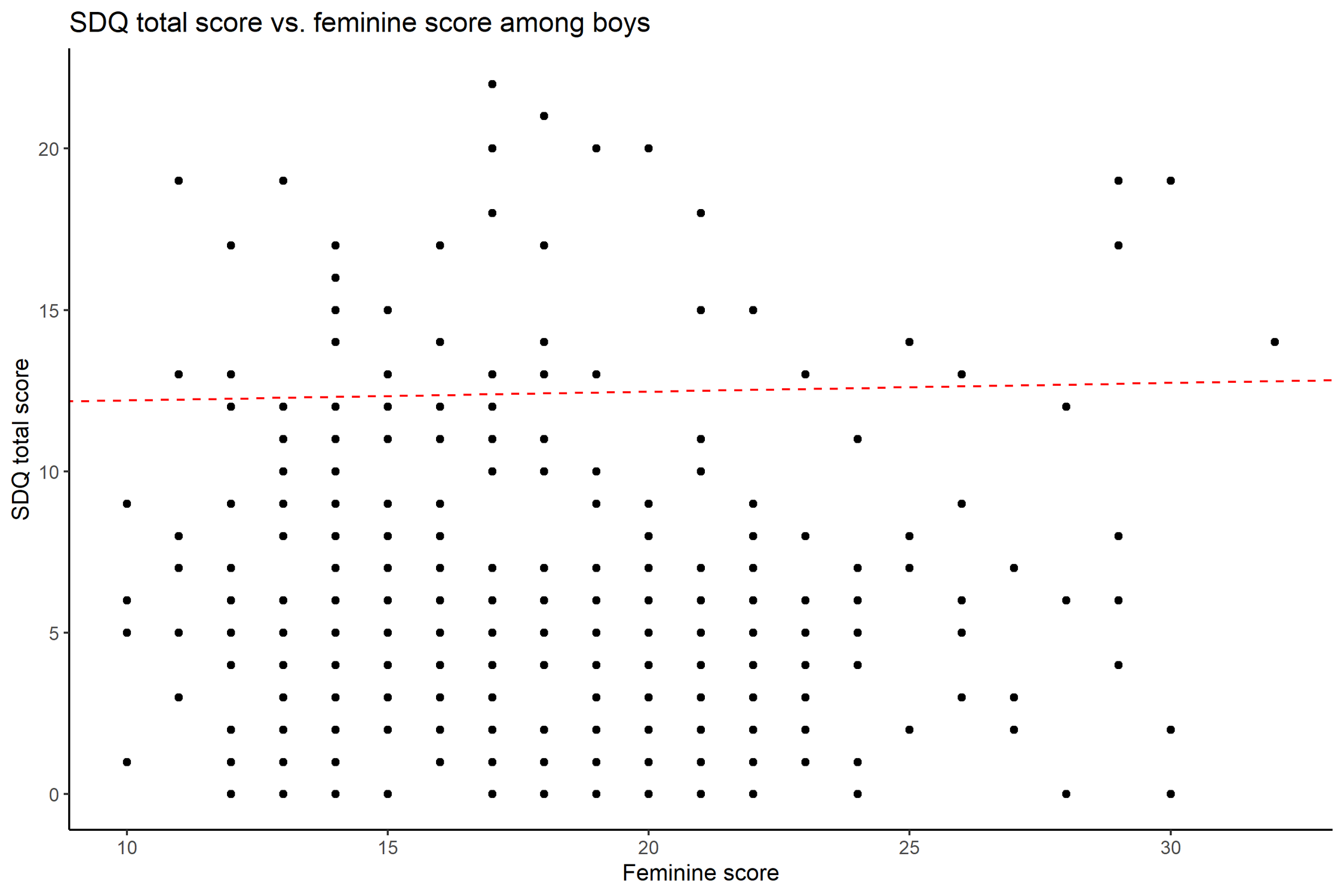

Supplement: S2 Fig — (TIF) [file pone.0308605.s006.tif]

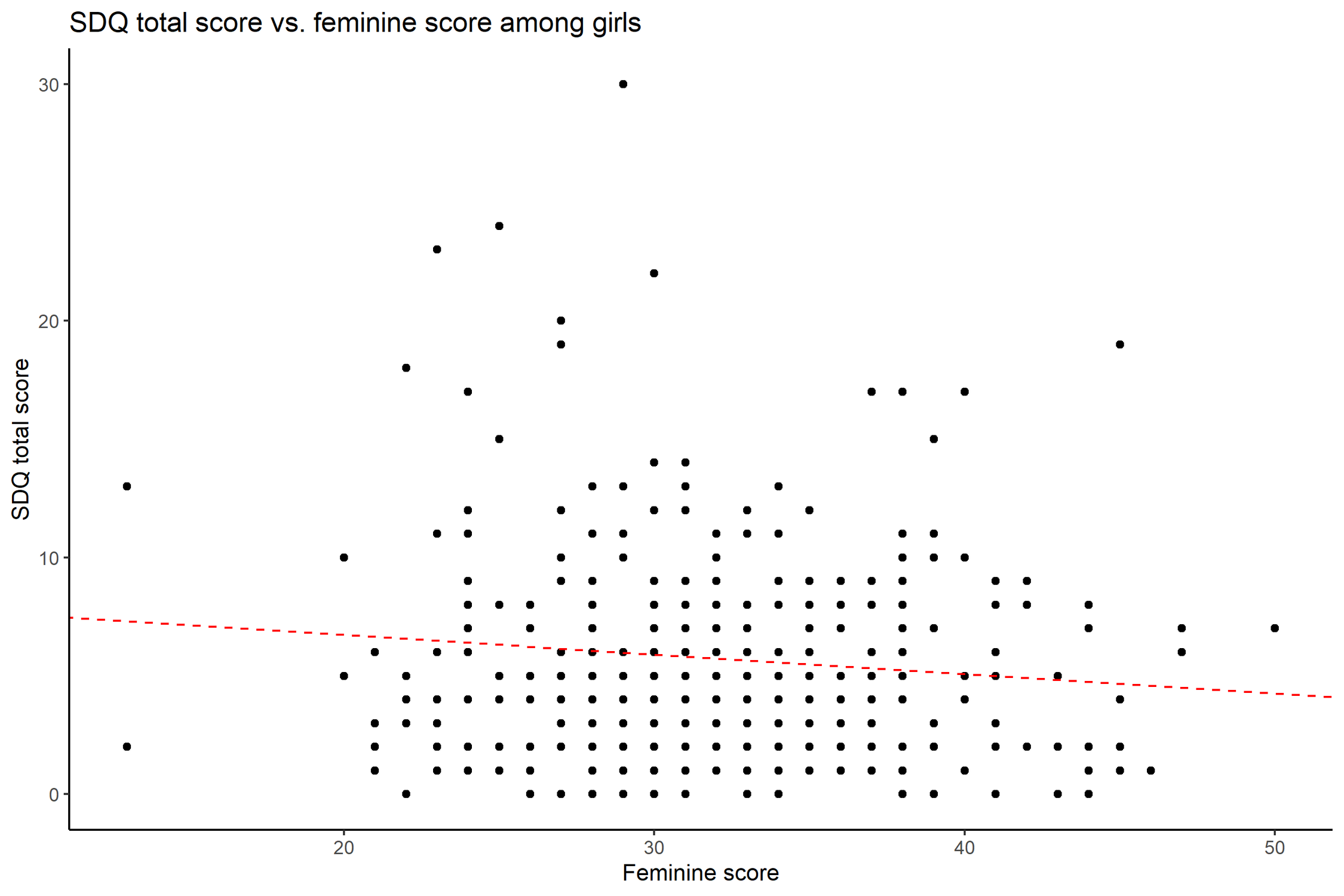

Supplement: S3 Fig — (TIF) [file pone.0308605.s007.tif]

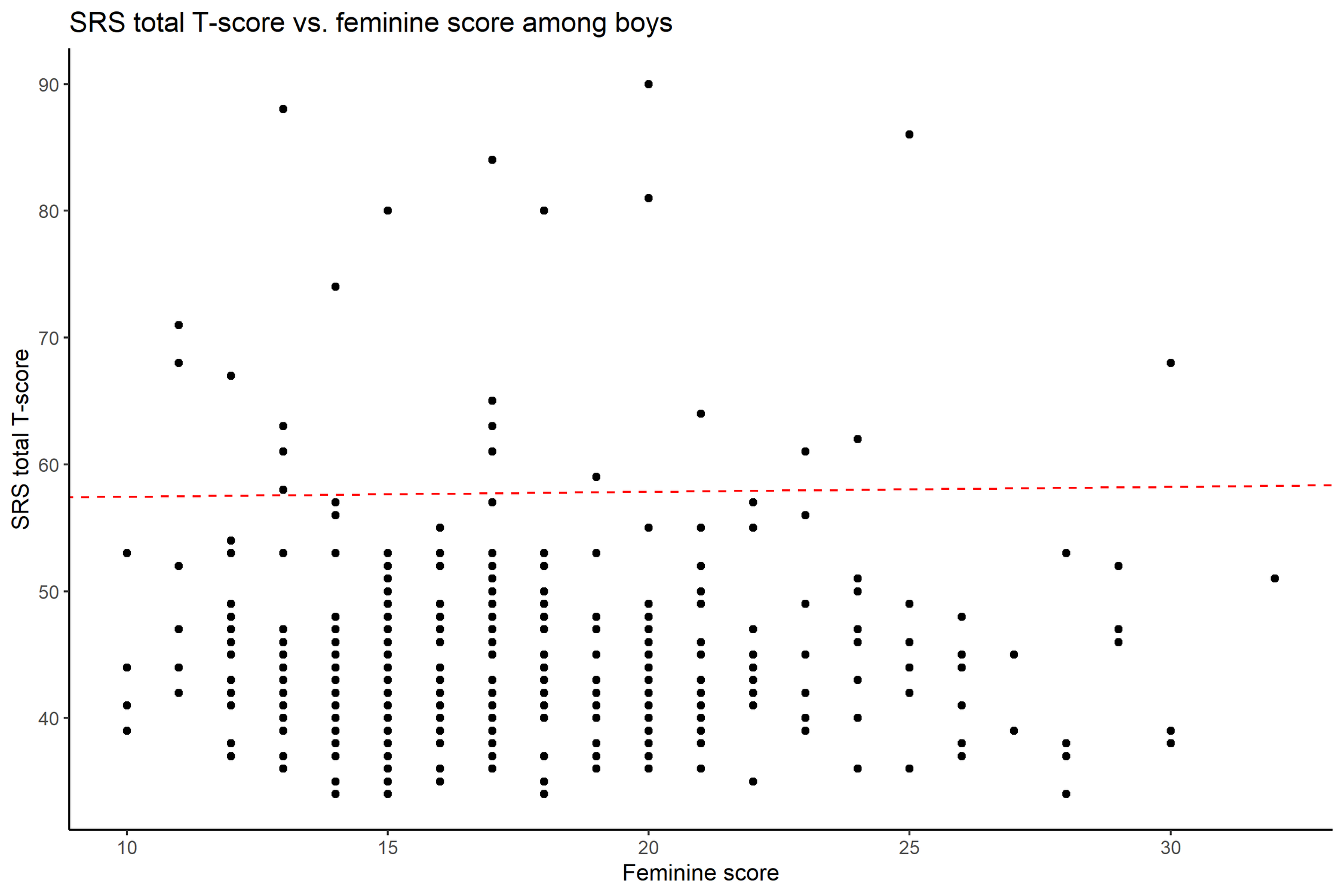

Supplement: S4 Fig — (TIF) [file pone.0308605.s008.tif]

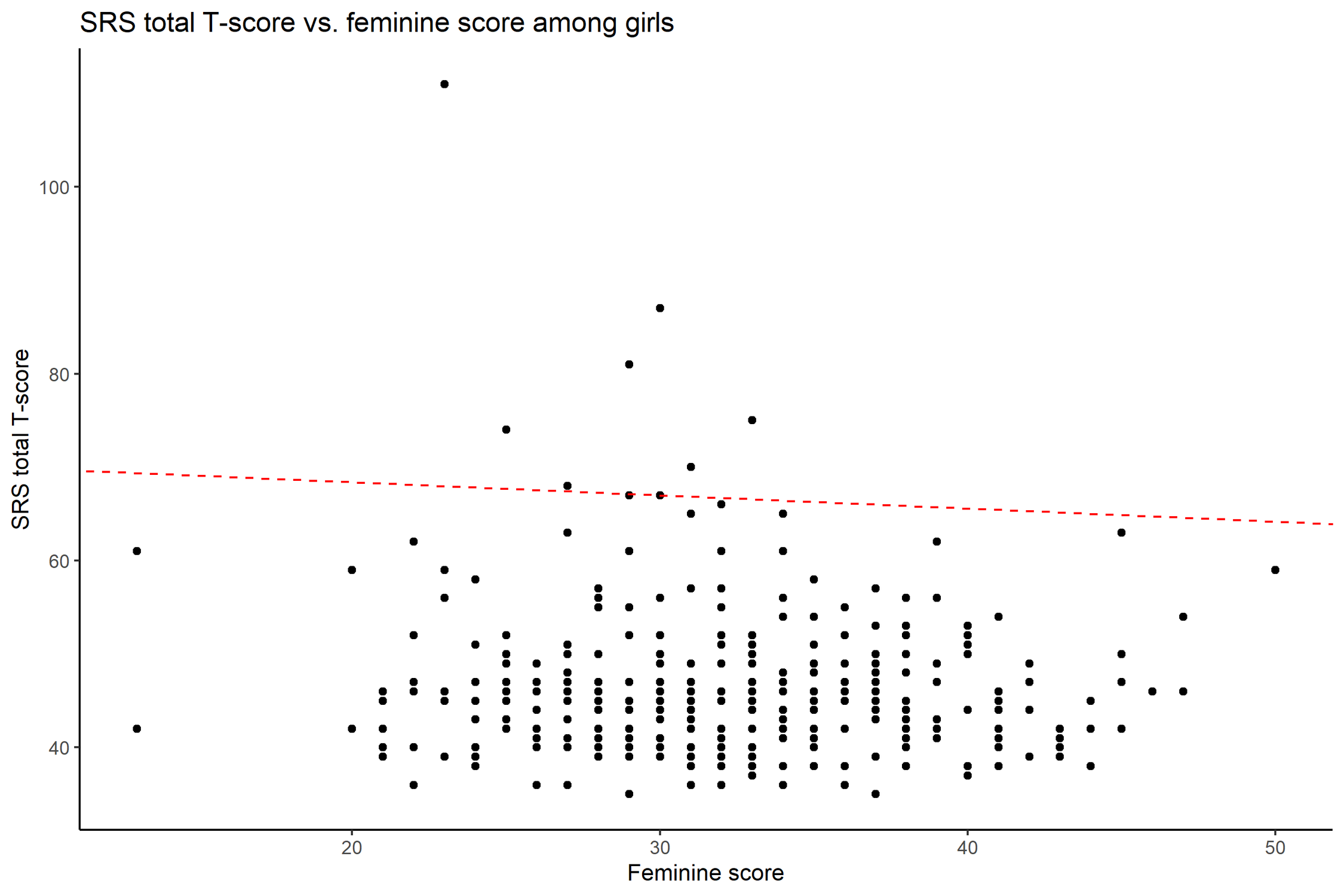

Supplement: S5 Fig — (TIF) [file pone.0308605.s009.tif]
