# Supplementary material for: Gender-specific play behavior in relation to autistic traits and behavioral difficulties at the age of seven in the SELMA study
Source: PLoS One. 2024 Aug 28;19(8):e0308605. doi: 10.1371/journal.pone.0308605 (PMC11355531; doi:10.1371/journal.pone.0308605)
Supplement: S1 Questionnaire — (DOCX) [file pone.0308605.s010.docx]

S1 Questionnaire. PSAI used in the SELMA study.

|  | Never | Almost never | Some-  times | Often | Very often |
| --- | --- | --- | --- | --- | --- |
| *Toys* |  |  |  |  |  |
| 1. Guns (or used objects as guns) |  |  |  |  |  |
| 2. Jewelry |  |  |  |  |  |
| 3. Tool set |  |  |  |  |  |
| 4. Action figures |  |  |  |  |  |
| 5. Dolls, doll’s clothes, or doll’s carriage |  |  |  |  |  |
| 6. Trains, cars or airplanes |  |  |  |  |  |
| 7. Swords (or used objects as guns) |  |  |  |  |  |
| 8. Tableware |  |  |  |  |  |
| *Activities* |  |  |  |  |  |
| 9. Playing house (e.g. cleaning, cooking) |  |  |  |  |  |
| 10. Playing with girls |  |  |  |  |  |
| 11. Pretending to be a female character (e.g. princess) |  |  |  |  |  |
| 12. Playing like police, soldier, or firefighter |  |  |  |  |  |
| 13. Fighting |  |  |  |  |  |
| 14. Climbing (e.g. climbing frames, trees, climbing walls) |  |  |  |  |  |
| 15. Playing at taking care of babies |  |  |  |  |  |
| 16. Showing interest in real cars, trains, or airplanes |  |  |  |  |  |
| 17. Dressing up in girlish clothes |  |  |  |  |  |
| *Characteristics* |  |  |  |  |  |
| 18. Likes to explore new places |  |  |  |  |  |
| 19. Likes rough and tumble play |  |  |  |  |  |
| 20. Shows interest in snakes, spiders, or insects |  |  |  |  |  |
| 21. Avoids getting dirty |  |  |  |  |  |
| 22. Likes pretty thing (e.g. flowers, glittering things) |  |  |  |  |  |
| 23. Avoid taking risks |  |  |  |  |  |
