# Supplementary material for: Gender-specific play behavior in relation to autistic traits and behavioral difficulties at the age of seven in the SELMA study
Source: PLoS One. 2024 Aug 28;19(8):e0308605. doi: 10.1371/journal.pone.0308605 (PMC11355531; doi:10.1371/journal.pone.0308605)
Supplement: S2 Questionnaire — (DOCX) [file pone.0308605.s011.docx]

S2 Questionnaire. The scale on parental attitudes toward play behavior used in the SELMA study.

|  | Absolutely not | Probably not | Maybe | Likely | Highly likely |
| --- | --- | --- | --- | --- | --- |
|  | | | | | |
| 1. Musical instruments |  |  |  |  |  |
| 2. Guns |  |  |  |  |  |
| 3. Jewelry |  |  |  |  |  |
| 4. Tool set |  |  |  |  |  |
| 5. Pens, crayons, paper |  |  |  |  |  |
| 6. Action figures |  |  |  |  |  |
| 7. Dolls, doll’s clothes, or doll’s carriage |  |  |  |  |  |
| 8. Books |  |  |  |  |  |
| 9. Trains, cars, or airplanes |  |  |  |  |  |
| 10. Swords (or used objects as guns) |  |  |  |  |  |
| 11. Tableware |  |  |  |  |  |
|  | | | | | |
| 1. Playing house (e.g. cleaning, cooking) |  |  |  |  |  |
| 2. Playing with girls |  |  |  |  |  |
| 3. Reading |  |  |  |  |  |
| 4. Drawing and painting |  |  |  |  |  |
| 5. Playing like a female character (e.g. princess) |  |  |  |  |  |
| 6. Playing like police, soldier, or firefighter |  |  |  |  |  |
| 7. Fighting |  |  |  |  |  |
| 8. Climbing (e.g. climbing frames, trees, climbing walls) |  |  |  |  |  |
| 9. Playing at taking care of babies |  |  |  |  |  |
| 10. Playing music |  |  |  |  |  |
| 11. Showing interest in real cars, trains, or airplanes |  |  |  |  |  |
| 12. Dressing up in girlish clothes |  |  |  |  |  |
