# Supplementary material for: Gender-specific play behavior in relation to autistic traits and behavioral difficulties at the age of seven in the SELMA study
Source: PLoS One. 2024 Aug 28;19(8):e0308605. doi: 10.1371/journal.pone.0308605 (PMC11355531; doi:10.1371/journal.pone.0308605)
Supplement: Attachment — Submitted filename: Response to Reviewers.docx [file pone.0308605.s012.docx]

Dear Dr. Pisula, Dr. Niedźwiecka and reviewers,

We appreciate the opportunity to review our manuscript. We believe that the manuscript improved with your ideas and suggestions and hope that we addressed them to your satisfaction. Please see our point-by-point responses below.

Best regards,

Marlene Stratmann

**Journal Requirements**:

When submitting your revision, we need you to address these additional requirements.

1. Please ensure that your manuscript meets PLOS ONE's style requirements, including those for file naming. The PLOS ONE style templates can be found at
<https://journals.plos.org/plosone/s/file?id=wjVg/PLOSOne_formatting_sample_main_body.pdf> and <https://journals.plos.org/plosone/s/file?id=ba62/PLOSOne_formatting_sample_title_authors_affiliations.pdf>

*Response: Thank you for reminding us of the requirements. We have updated the manuscript and supporting information according to the guidelines.*

2. Thank you for stating the following financial disclosure: "This work received funding from the European Union’s Horizon 2020 research and innovation program under grant agreement no. 634880, EDC-MixRisk". Please state what role the funders took in the study.  If the funders had no role, please state: "The funders had no role in study design, data collection and analysis, decision to publish, or preparation of the manuscript." If this statement is not correct you must amend it as needed. Please include this amended Role of Funder statement in your cover letter; we will change the online submission form on your behalf.

*Response: We have updated the cover letter accordingly.*

3. We note that you have indicated that there are restrictions to data sharing for this study. For studies involving human research participant data or other sensitive data, we encourage authors to share de-identified or anonymized data. However, when data cannot be publicly shared for ethical reasons, we allow authors to make their data sets available upon request. For information on unacceptable data access restrictions, please see<http://journals.plos.org/plosone/s/data-availability#loc-unacceptable-data-access-restrictions>.

Before we proceed with your manuscript, please address the following prompts:

a) If there are ethical or legal restrictions on sharing a de-identified data set, please explain them in detail (e.g., data contain potentially identifying or sensitive patient information, data are owned by a third-party organization, etc.) and who has imposed them (e.g., a Research Ethics Committee or Institutional Review Board, etc.). Please also provide contact information for a data access committee, ethics committee, or other institutional body to which data requests may be sent.

*Response: We have added contact information to the data availability statement in the submission system in case someone wants to request the data. We also explained that there are legal and ethical restrictions according to the General Data Protection Regulation, the Swedish law, the Swedish Data Protection Act, and the Swedish Ethical Review Act, as data can be used to potentially identify study participants and the data contains sensitive information.*

b) If there are no restrictions, please upload the minimal anonymized data set necessary to replicate your study findings to a stable, public repository and provide us with the relevant URLs, DOIs, or accession numbers. Please see<http://www.bmj.com/content/340/bmj.c181.long> for guidelines on how to de-identify and prepare clinical data for publication. For a list of recommended repositories, please see<https://journals.plos.org/plosone/s/recommended-repositories>. You also have the option of uploading the data as Supporting Information files, but we would recommend depositing data directly to a data repository if possible.

Please update your Data Availability statement in the submission form accordingly.

*Response: Restrictions apply to data availability; please refer to the previous comment.*

4. Please include captions for your Supporting Information files at the end of your manuscript, and update any in-text citations to match accordingly. Please see our Supporting Information guidelines for more information:<http://journals.plos.org/plosone/s/supporting-information>.

*Response: Thank you for bringing our attention to this. We updated the in-text citations and the captions according to the guidelines.*

**Additional Editor Comments**:

5. The article was thoroughly reviewed by two reviewers who made essential comments. They both appreciated the merit, although their opinions on the value of this work varied. Both reviewers pointed out several weaknesses in the paper, precisely suggesting specific changes. I fully agree with Reviewer 2 that the discussion section seems superficial and poorly theoretically grounded. The suggestion to refer to a paper by Hull et al. (2022) is also very valuable. Because the study has some strengths, its results are worth presenting, and the paper can be significantly improved according to the reviewers' comments, my decision is a major revision.

*Response: Thank you so much for your evaluation. All the comments have proven helpful and we changed the manuscript to the best of our ability to hopefully make the discussion more theoretically founded and deeper. We have also included the study by Hull et al. (2022), as it is perfectly connected to our research.*

**Reviewer # 1**

Review of the article Gender-specific Play in Relation to autistic traits and Behavioral Difficulties at the Age of Seven in the SELMA study

Thank you for the opportunity to review this manuscript.

The article reports the results of a study examining the relationship between feminine vs. masculine types of play in children and their autistic traits and problem behaviors. The feminine/masculine play scores were used as a proxy for gender nonconformity. Assessments were based on parental reports. The design of the study was correlational. The Authors found gender-specific differences in the effects they observed. Overall, I think this work may make a valuable contribution, but it requires improvement. I list my concerns below.

 Comments

Introduction

1.       Line 76 Please define the term “gender diversity.”

*Response: We included the definition of gender diversity and it now is: “Still, the proportion of transgender and gender diverse persons, i.e., people whose gender identities and/or expressions are different from sociocultural norms, in the general population is estimated to be between 0.02 and 8.4% [7].”*

2.       Line 104 There seems to be a problem with this sentence. Did you mean “that shed light” instead of “to shed light”?

*Response: Thank you for pointing out this error. We have updated the sentence according to your suggestions.*

 Results

3.       Description of the results and Table 1 Please provide the effect sizes where applicable.

*Response: Thank you for this comment. As we only present the descriptive characteristics of our study population and comparisons of those characteristics between girls and boys are not our main research questions, we decided not to show effect sizes or p-values in Table 1. However, we present effect sizes and p-values for PSAI, SRS, and SDQ in Supplementary Table 1 considering those comparisons are relevant to the main research questions and may help readers to interpret the main results better. We added a sentence to the method section to point to the supplementary material.*

4.       Regarding the result indicating higher SRS scores in girls than boys, please give a possible explanation in the Discussion section. Please clarify whether subscale scores in SES and SDQ subscales differed significantly in boys and girls.

*Response: Thank you for your comment. As explained in the previous comments, p-values are now presented in Supplementary Table 1. The result indicating higher SRS scores among girls compared with boys is not significant. Since we used standardized SRS scores, which are adjusted for sex differences, this marginal non-significant difference should not be a concern in a population-based study sample. For this reason, we did not elaborate on that finding in the discussion section.*

5.       Results of regression analyses Please report collinearity statistics. Please provide scatterplots to demonstrate a linear association between variables, which is the key assumption for conducting linear regression. Please discuss the effect sizes.

*Response: As we examined several associations with linear regression analysis, we exemplified collinearity scatterplots for some of the associations in Supplementary Figures 2-5. Collinearity statistics are provided in the extra file for regression analysis along with the full model fit statistics.
Based on your suggestions, we put more emphasis on the effects sizes in the discussion section as follows: “All abovementioned associations display small effect sizes considering the scales of dependent variables, which is expected within a non-clinical study sample.”* And further: *“Having a population-based study sample utilizing symptoms might have led to non-significant results in boys and partly in girls and small effect sizes in general.”*

6.       Tables 2 and 3 are cut.

Response: *We apologise for this. According to the journal guidelines regarding size of tables we referred to this rule: “Do not split your table or otherwise try to make the table appear within the manuscript margins if it does not fit on one page. In Word, tables that run off of the manuscript page can be seen using Draft View. In the PDF version of the published article, very wide tables may be printed sideways, and long tables may span more than one page.” We have changed this to make the tables readable in non-draft view in Word.*

 Discussion

7.       Line 294 Please provide references.

*Response: We included references following your suggestion.*

8.       Lines 302-304 I think that drawing such firm conclusions based on null results (here: non-significant associations) is an overstatement.

*Response: We toned our statement down as follows: “Similar to those studies, we did not observe any significance regarding autistic traits in boys.”*

9.       Lines 314-325 How are these considerations related to your study?

*Response: Thank you for your comment. We adjusted that section and emphasized its relation to our study as follows:*

*“In recent years, the binary framework of sex and gender has also been started to be disregarded in neurobiology. For instance, the mosaic brain theory has been proposed, which claims that biological sex affects the brain of both females and males through several mechanisms and leads to “femaleness” or “maleness” in different features of the brain [49]. A recent study by Rodin et al. identified specific mosaics in the brains of people with ASD, which implies a less binary interpretation of sex and gender in autism [50]. These findings align with the study by Hull et al., which showed less gendered developmental trajectories of play behavior among children with ASD in comparison to the ones without ASD [16].  Even though our results on autistic traits might also signal less gender-specific play for girls, we did not observe such a finding for boys. Using scales as PSAI does not allow for exploring play behavior beyond the binary perspective, and future research should also focus on gender-neutral play respecting the accumulating evidence on play behavior in ASD.”*

10.   Lines 326-341 Here, I am confused, as the study involved the general population and did not really focus on a sample of children with a diagnosis of ASD.

*Response: In that paragraph, we aim to present potential explanation not only for GD and ASD but also for GNC and autistic tratis. We adjusted the text not to lead any confusion and put more emphasis on the fact that we only investigated symptom dimensions: “Hypotheses attempting to delineate the link between GNC/GD and autistic traits/ASD are not limited to biological ones, and social and psychological factors have also been proposed [26].” And further: “While potential explanations have mainly been proposed for clinical conditions, it is important to note that our findings on symptoms cannot be generalized to clinical diagnoses…”*

11.   Lines 363-365 Clearly, the directionality cannot be determined in a correlational study like this one. Would it not be better to use the term “dependent variable” instead of “outcome” throughout the article?

*Response: Thank you for your suggestion. We replaced the term “outcome variable” with “outcome variable” throughout the text.*

12.   Please discuss the use of parental evaluation of play type as a proxy for gender nonconformity. Is this a sufficient indicator?

*Response: We improved our statement in the discussion section concerning the potential limitations of using a parent-reported play behavior as a proxy for gender nonconformity: “Third, we only focused on a single domain of GNC and used play behavior as a proxy, but GNC can certainly be expressed in various ways. Yet, play behavior is emphasized in different diagnostic criteria for GD or gender incongruence in children, which puts its relevance forward within this context. Fourth, all independent and dependent variables were collected through parent-reported scales. Parents might have different tendencies to over- or underestimate their children's behavior, which might have partly resulted in non-differential misclassification of independent and dependent variables.”*

13.   Overall, the results of the study have not been adequately and thoroughly discussed. For instance, “social and psychological factors” (line 327) have been vaguely mentioned but not addressed regarding possible mechanisms of gender differences in the present study.

*Response: Thank you for your suggestion. We aimed to improve the discussion section following your comments. Specifically, we elaborated on social and psychological factors as follows: “Hypotheses attempting to delineate the link between GNC/GD and autistic traits/ASD are not limited to biological ones, and social and psychological factors have also been proposed [26]. Individuals with ASD exhibit difficulties to conform to social norms and expectations and this aspect of ASD has been proposed to be linked to the development of GNC and GD [51]. Different gender identities have also been stated to be parts of varied perceptions of one’s whole identity among persons with ASD [52]. A few researchers have also hypothesized GNC and symptoms of GD as preoccupations and obsessive thoughts in relation to ASD [53, 54], but these hypotheses seem to be limited with case reports. Various biological, social and psychological factors might contribute to the high co-occurrence of GD and ASD, and this might extend to our results on associated symptoms among girls…”*

14.   Supplementary Figure 1 Please add an annotation explaining what “I” means.

*Response: We added an explanation in the figure, which is: “The green node indicates the independent variable, the blue node the dependent variable. The red nodes indicate potential confounding factors.” The “I” in the DAG does not mean anything specific but is a choice from the developers of dagitty.net to indicate the dependent variable. We hope that the added explanation is sufficient.*

**Reviewer #2**

My thanks for the opportunity to read this paper exploring gender-specific play and the relationship with autistic traits and problem behaviours. The authors found gender-atypical play was associated with higher autistic traits in girls, and with subtypes of behavioural difficulties in both boys and girls. Although this paper does have strengths, such as exploring these associations in a large, population-based sample, there are several limitations which for me detract from its impact. Notably, the paper is missing engagement with a recent study exploring a very similar research question, which raised interesting hypotheses around the development of gender-specific play, and which could extent the discussion of the present paper. Additionally, the discussion section felt quite descriptive to me and did not really address any theories as to why the observed differences/lack thereof may have been observed. In particular I would have liked to see more consideration of how autistic traits and problem behaviours might be related to each other or distinct, and how gender-specific play interacts with both of these in childhood. The paper requires some reworking to make a clear contribution to current understanding.

Comments:

1.       Abstract – ‘higher composite scores’ is quite hard to interpret for someone who doesn’t know the measure – could this be described more precisely (e.g. “increased masculine/decreased feminine play”)?

*Response: We agree that this can be confusing. We have clarified this sentence and it now reads “Higher composite play behavior scores (indicating either increased masculine or decreased feminine play behavior) were associated with increased autistic trait scores in girls [...]”.*

2.       What exactly do the conclusions mean for clinical settings or for our understanding of gender development? It’s not clear what the impact of these findings would be from the abstract.

*Response: Thank you for pointing this out. We have added a sentence and the conclusion now is “This study suggests a link between gender nonconforming play behavior and autistic traits as well as behavioral difficulties among children in a non-clinical population, which calls attention to the necessity of supporting children with gender nonconformity from early ages.”.*

3.       Funding – please supply more information regarding who the funding was awarded to and whether the funders played a role in any stage of the study.

*Response: We have updated this in the cover letter, and it now reads as follows “This work received funding from the European Union’s Horizon 2020 research and innovation program under grant agreement no. 634880, EDC-MixRisk and the funders had no role in study design, data collection and analysis, decision to publish, or preparation of the manuscript”. The information will be included in the published version of this manuscript.*

Introduction

4.       It is good that the authors distinguish between gender non-confirming and gender dysphoria. However I’d like to see some more consideration of how gender-specific play fits within these categories – how do these authors define this, and why is gender-specific play an appropriate proxy for gender non-confirming behaviour in this sample?
 There is a relatively recent study (Hull, Heuvelman et al., 2022; DOI: 10.1177/13623613221139373) that explored precisely this question of gender-specific play and the association with autism within longitudinal population-level data. I think it is important that the authors engage with this study, particularly the age-related findings for boys, in both the introduction and discussion of this article.

*Response: We elaborated on what gender-specific paly behavior is an appropriate proxy in the discussion section as follows:* *“Third, we only focused on a single domain of GNC and used play behavior as a proxy, but GNC can certainly be expressed in various ways. Yet, play behavior is emphasized in different diagnostic criteria for GD or gender incongruence in children, which puts its relevance forward within this context.”*
*Thank you for bringing this article to our attention. We have included this article in the introduction as well as the discussion and made it one of the key studies to compare our findings to, as many aspects are similar in these two studies.*

 Methods

5.       Some more detail about the SDQ would be helpful, such as describing the different subscales and how they are interpreted – it might be helpful, for instance, to note that the prosocial behaviour subscale is scored in reverse to the others, with higher scores representing fewer behavioural difficulties.

*Response: Thank you for your suggestion, we agree that more information could be useful. Regarding the reversely scored prosocial behaviour subscore, there is information as “Each subscore can range from 0 to 10 points, where a higher score indicates more difficulties, except for the prosocial behavior subscore that is reversely scored, where a higher score indicates higher prosocial behavior” in Methods. For the different subscores we added more information to the existing sentences to make it clearer what these subscores indicate. The sentence is now as follows “Each difficulty subscore (emotional symptoms, conduct problems, hyperactivity and inattention and peer relationship problems) can range from 0 to 10 points, where a higher score indicates more difficulties, except for the prosocial behavior subscore that is reversely scored, where a higher score indicates higher prosocial behavior”*

6.       Were the same confounders proposed for both autistic trait and problem behavior outcomes? What was the rationale behind this?

*Response: Indeed, the same set of confounders was proposed for both autistic traits and behavioral outcomes. The rationale behind this was that many mechanisms could be very similar. Hence, we chose to have this minimal set of adjustments to not open any potential backdoor paths when adjusting for colliders.*

Results

7.      Is there any information on how representative the participants were of the entire study population? E.g. did included versus excluded/missing data participants differ on any key variables or characteristics?

*Response: We included the comparisons of descriptive characteristics between included and excluded participants in the supplementary material, table 1.*

8.       Tables 2 and 3 were cut off for me and so I could not read all results. Please could these be reformatted to landscape orientation or presented on separate pages.
*Response: We apologise for this. According to the journal guidelines regarding size of tables we referred to this rule: “Do not split your table or otherwise try to make the table appear within the manuscript margins if it does not fit on one page. In Word, tables that run off of the manuscript page can be seen using Draft View. In the PDF version of the published article, very wide tables may be printed sideways, and long tables may span more than one page.” We have changed this to make the tables readable in non-draft view in Word.*

9.       Please also present the full model fit statistics for each model somewhere (e.g. in supplementary material).

*Response: We have now also uploaded a file that is named “Regression analyses where full model fit statistics are shown as well as collinearity statistics.*

 Discussion

10.   What do these findings actually mean? So far the discussion seems very descriptive with little consideration of how this relates to either theory around child development, or supporting children with autistic traits or behavioural difficulties. In particular, I’d like to see more discussion of why different patterns of associations were found in boys and girls – which factors might be driving this?
Again I think it would be helpful to refer to the paper by Hull et al. (2022) which engages with the Extreme Male Brain theory and other theories of gender development in autism, and advocates for a developmental approach to these theories. In addition, this paper found that the age at which play behaviours are measured might impact gender typicality for boys; this could be discussed here.

*Response: Thank you so much for your suggestions. We revised the discussion section by providing more insights into developmental approaches, potential explanations for gender differences and timing of play behavior assessment:* *“We present different patterns of associations between gender-specific play and autistic traits among girls and boys, which should be interpreted in connection to child development. Gender stereotypes become rigid between ages three and five years [42], which extends to gender-specific play [36], and diminish later on. Gender-specific play changes during childhood and the timing of assessment has an impact on these changes, as demonstrated by Hull et al. [16]. In our study, play behavior was evaluated at the age of seven, which may have contributed to capturing reduced gender-specific play and, consequently, non-significant results. Additionally, boys are suggested to exhibit less gender variation in behavior compared with girls due to facing more social pressure to adhere to gender norms [43]. This tendency might also apply to gender-specific play behavior in this current study, potentially contributing to non-significant results among boys.”*

*Following your suggestions, we engaged our discussion with the study by Hull et al., which provides important insights for the interpretation of our results:*

*“On the other hand, Hull et al. showed that boys with ASD engaged in less gendered play compared to boys without ASD, while there was no significant difference in girls’ play based on ASD [16]”*

*“Gender-specific play changes during childhood and the timing of assessment has an impact these changes, as demonstrated by Hull and colleagues [16]”*

*“These findings align with the study by Hull et al., which showed less gendered developmental trajectories of play behavior among children with ASD in comparison to the ones without ASD [16].”*

11.   The authors refer very briefly to the idea of moving beyond a binary concept of sex and gender, but could expand upon this. I wonder again how much the binary measure used (PSAI) plays a role in this – it is not possible to look at neutral/agender behaviours if all play behaviours are categorised as only male or female. How would the authors recommend research explore beyond binary sex/gender in the future?
 I didn’t quite follow the argument made in the first paragraph of page 17. Are the authors suggesting that the SDQ and SRS are measuring the same construct? If so, was this autistic traits or problem behaviours? And why were all the results presented separately?

*Response: Thank you for your comments. We revised the part on the idea of moving beyond a binary concept of sex and gender and elaborated on future research directions as follows:*

*“In recent years, the binary framework of sex and gender has been started to be disregarded in neurobiology. For instance, the mosaic brain theory has been proposed, which claims that biological sex affects the brain of both females and males through several mechanisms and leads to “femaleness” or “maleness” in different features of the brain [49]. A recent study by Rodin et al. identified specific mosaics in the brains of people with ASD, which implies a less binary interpretation of sex and gender in autism [50]. These findings align with the study by Hull et al., which showed less gendered developmental trajectories of play behavior among children with ASD in comparison to the ones without ASD [16]. Even though our results on autistic traits might also signal less gender-specific play for girls, we did not observe such a finding for boys. Using scales as PSAI does not allow for exploring play behavior beyond the binary perspective, and future research should also focus on gender-neutral play respecting the accumulating evidence on play behavior in ASD.”*

*We suggest that SRS and SDQ measure different constructs even though they might overlap to some extent. We improved the paragraph you referred not to lead any confusion as follows:*

*“...These are indeed noteworthy statements, and to overcome the potential misclassification of behavioral outcomes, the dependent variables consisted of two different scales in our study (SRS and SDQ), as it has been recommended in the literature [55]. While autistic traits might potentially lead to some behavioral difficulties, examining two sets of symptom clusters helps distinguish the relationships with GNC…”*

*We prefer presenting results separately since they are different symptom clusters. We also hope readers follow easier with this structure.*
